# Iatrogenic tension pneumothorax in children: two case reports

**DOI:** 10.4076/1752-1947-3-7390

**Published:** 2009-06-30

**Authors:** Juan Mayordomo-Colunga, Corsino Rey, Alberto Medina, Andrés Concha

**Affiliations:** 1 Pediatric Intensive Care Unit, Department of Pediatrics, Hospital Universitario Central de Asturias, University of Oviedo, Oviedo, Spain

## Abstract

**Introduction:**

Two cases of iatrogenic tension pneumothorax in children are reported.

**Case presentations:**

Case 1: A 2-year-old boy with suspected brain death after suffering multiple trauma suddenly developed intense cyanosis, extreme bradycardia and generalized subcutaneous emphysema during apnea testing. He received advanced cardiopulmonary resuscitation and urgent bilateral needle thoracostomy. Case 2: A diagnostic-therapeutic flexible bronchoscopy was conducted on a 17-month-old girl, under sedation-analgesia with midazolam and ketamine. She very suddenly developed bradycardia, generalized cyanosis and cervical, thoracic and abdominal subcutaneous emphysema. Urgent needle decompression of both hemithoraces was performed.

**Conclusion:**

In techniques where gas is introduced into a child's airway, it is vital to ensure its way out to avoid iatrogenic tension pneumothorax. Moreover, the equipment to perform an urgent needle thoracostomy should be readily available.

## Introduction

Pneumothorax can be classified as spontaneous, traumatic or iatrogenic [1]. The most frequent cause of iatrogenic pneumothorax is transthoracic pulmonary biopsy, but it also may appear as a complication of many other procedures [2]. Iatrogenic pneumothorax has been reported in up to 3% of adult patients admitted to intensive care units (ICU) [3]. Its management can be either conservative or aggressive depending on the patient's clinical status [4] and the size of the pneumothorax (a volume higher than 20% of pleural space indicates the need for pleural drainage [5]). For a tension pneumothorax (TPT), immediate air evacuation must be performed. Although most TPTs are traumatic, they can occasionally have an iatrogenic cause.

## Case presentations

### Case 1

A 2-year-old boy was admitted to our pediatric ICU after suffering severe multiple trauma. He progressed to refractory intracranial hypertension within 24 hours, and clinical tests indicated brain death. After preoxygenation with 100% FiO_2_ for 1 minute, apnea testing was performed using a supplemental oxygen cannula (an 8-French nasogastric tube) at a 6 L/minute flow rate through a 4.5 mm inner diameter (ID) endotracheal tube (ETT). About 60 seconds later, the child suddenly developed intense cyanosis, severe bradycardia and massive subcutaneous emphysema. The supplemental oxygen cannula was immediately removed and urgent needle thoracostomy was performed on both hemithoraces. A large amount of air was evacuated from the right hemithorax, and some from the left one. A chest tube was placed on the right side. Chest compressions and three doses of adrenaline were also applied, and the patient regained stability.

Chest X-ray performed after the acute situation showed pneumoperitoneum, subcutaneous emphysema and right pneumothorax, with a chest tube on the right side. According to the family's wishes, organ donation was performed 2 days later.

### Case 2

A 17-month-old girl presented with persistent atelectasis of her left lower lobe as a consequence of complicated pneumonia at the age of 8 months. A diagnostic-therapeutic bronchoscopy was performed. The girl was symptom-free, with 100% oxygen saturation while breathing room air before undergoing the procedure. A 3.2 mm ID flexible bronchoscope was used, under unconscious sedation-analgesia with intravenous midazolam and ketamine. After two unsuccessful attempts due to desaturation, supplemental oxygen was administered through the bronchoscope suction channel at 5 L/minute flow rate. Ninety seconds later, the girl abruptly developed bradycardia, cyanosis and cervical, thoracic and abdominal subcutaneous emphysema. With the suspicion of TPT, an urgent needle decompression was performed on both hemithoraces, and the patient was subsequently intubated. Air escape was noted and chest tubes were inserted on both sides. The girl recovered to a stable situation and was maintained on mechanical ventilation for 72 hours, with no other complications.

**Figure 1:**
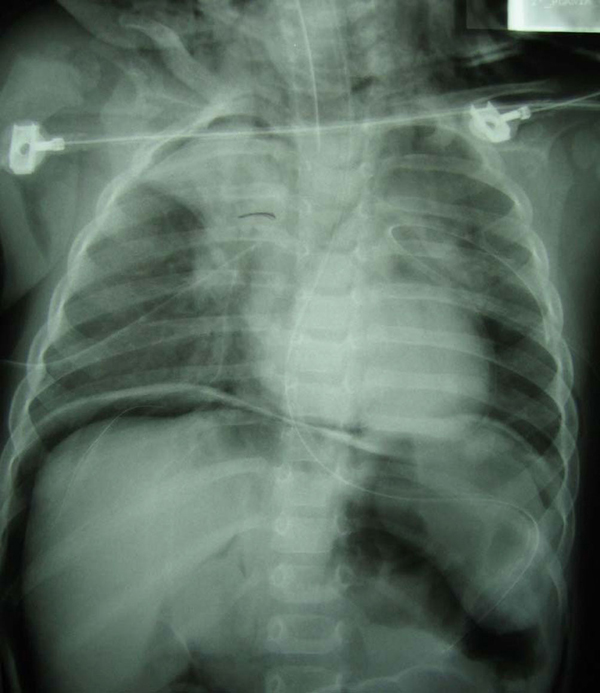
**Thorax-abdomen radiography showing pneumopericardium, pneumoperitoneum, subcutaneous emphysema and right pneumothorax, with right upper lobe atelectasis**. Chest tubes in both hemithoraces.

Chest X-ray revealed pneumopericardium, pneumoperitoneum, subcutaneous emphysema and right pneumothorax, with a chest tube on the right side and right upper lobe atelectasis (Figure 1).

## Discussion

TPT is lethal without immediate treatment. Therefore, it should be recognized promptly during the patient's clinical evaluation because there is no time for performing a chest X-ray. In ventilated patients, the onset of TPT is always rapid and it immediately produces a progressive drop of oxygen saturation and cardiac output, an increase in ventilation pressures, ipsilateral chest hyper-expansion, hypomobility and hypoventilation [6]. It is worth noting that any pneumothorax can turn into a TPT if positive pressure is applied.

The most frequent pneumothorax findings in a conscious patient are respiratory distress, chest pain, tachycardia and ipsilateral hypoventilation, according to Leigh-Smith's review [6]. When symptoms and signs are not clear and the patient's situation is not critical, chest X-ray can be very helpful. In TPT, as in our two cases, abrupt presentation and impending cardiac arrest demand immediate needle decompression.

The same mechanism was responsible for TPT development in our two reported cases. Supplemental oxygen was administered in both cases, and the air outflow was partially blocked, so that air entry exceeded air exit. This was the reason why the sequence of lung hyperinflation, tension pneumothorax, pneumomediastinum and subcutaneous emphysema occurred [7]. A case reported by Burns and Russell during apnea testing had similar manifestations, but the main production mechanism was a perforation of the distal trachea or left mainstem bronchus by the oxygen cannula [8].

In both our cases, the external diameters of the endotracheal cannula and of the bronchoscope should be markedly smaller than the ID of the ETT or the child's trachea. The appropriate oxygen flow in both techniques is not known, but it has been reported that this should not exceed 6 L/minute in adults and even less in small children during apnea testing [9].

When using an endotracheal catheter to perform an apnea test, the tip should never be introduced beyond the tip of the ETT and should never wedge against any structure [9]. An alternative to the oxygen cannula could be the use of a T-piece system, bulk diffusion and continuous positive airway pressure (CPAP) (Table 1), which would markedly reduce the risk of TPT [8,10].

**Table 1:** Preventive measures to avoid tension pneumothorax during apnea testing

• Make sure that the external diameter of the endotracheal cannula is markedly smaller than the inner diameter of the endotracheal tube.
• Do not ever introduce the endotracheal cannula tip beyond the tip of the endotracheal tube. It should never wedge against any structure.
• Oxygen flow should not exceed 6 L/minute in adults and even less in small children.
• T-piece systems, bulk diffusion, and continuous positive airway pressure can be used to provide supplemental oxygen instead of inserting an oxygen cannula into the endotracheal tube.

Oxygen insufflation through the suction channel of the bronchoscope is used to defog the lens, clear secretions and increase the fraction of inspired oxygen [11,12]. TPT is a rare complication in this technique, but the technique is considered to be safe when performed without the presence of an ETT. Moreover, no TPT was reported in de Blic's large patient series [13]. If mild hypoxemia occurs, oxygen might be provided using nasal prongs or a Venturi mask, or CPAP can be used [14,15] (Table 2).

**Table 2:** Preventive measures to avoid tension pneumothorax during flexible bronchoscopy on a spontaneously breathing patient

• Always use a bronchoscope diameter markedly smaller than inner diameter of the patient's trachea.
• Use nasal prongs, a Venturi mask or non-invasive positive airway pressure ventilation to avoid or treat hypoxemia, instead of using the bronchoscope suction port to provide supplemental oxygen.
• If oxygen administration through the suction port is needed, use low-oxygen flow.

## Conclusion

These cases highlight the importance of ensuring that air escape is available to avoid air trapping when performing techniques which introduce air into a patient's airway. This is particularly significant in young children. It is necessary to be aware of the risk of TPT and to have equipment readily available to perform an urgent needle thoracostomy.

## Abbreviations

CPAP: continuous positive airway pressure; ICU: intensive care unit; ID: inner diameter; ETT: endotracheal tube; TPT: tension pneumothorax.

## Consent

Written informed consent was obtained from the patient's parents for publication of this case report and any accompanying images. A copy of the written consent is available for review by the Editor-in-Chief of this journal.

## Competing interests

The authors declare that they have no competing interests.

## Authors' contributions

AM and AC were responsible for the diagnosis and treatment of the patients. CR and JMC performed the literature research and drafted the manuscript, which was read and approved by all authors in its final version.
